# Getting closer to social interactions using electroencephalography in developmental cognitive neuroscience

**DOI:** 10.1016/j.dcn.2024.101391

**Published:** 2024-05-14

**Authors:** Yvette Grootjans, Anita Harrewijn, Laura Fornari, Tieme Janssen, Ellen R.A. de Bruijn, Nienke van Atteveldt, Ingmar H.A. Franken

**Affiliations:** a Department of Psychology, Education and Child Studies, Erasmus University Rotterdam, the Netherlands; b Department of Clinical, Neuro, and Developmental Psychology & Institute LEARN!, Vrije Universiteit Amsterdam, the Netherlands; c Department of Clinical Psychology, Leiden University, the Netherlands

**Keywords:** Electroencephalography, Hyperscanning, Social interactions, Mobile EEG, Ecological validity, Real-life settings

## Abstract

The field of developmental cognitive neuroscience is advancing rapidly, with large-scale, population-wide, longitudinal studies emerging as a key means of unraveling the complexity of the developing brain and cognitive processes in children. While numerous neuroscientific techniques like functional magnetic resonance imaging (fMRI), functional near-infrared spectroscopy (fNIRS), magnetoencephalography (MEG), and transcranial magnetic stimulation (TMS) have proved advantageous in such investigations, this perspective proposes a renewed focus on electroencephalography (EEG), leveraging underexplored possibilities of EEG. In addition to its temporal precision, low costs, and ease of application, EEG distinguishes itself with its ability to capture neural activity linked to social interactions in increasingly ecologically valid settings. Specifically, EEG can be measured during social interactions in the lab, hyperscanning can be used to study brain activity in two (or more) people simultaneously, and mobile EEG can be used to measure brain activity in real-life settings. This perspective paper summarizes research in these three areas, making a persuasive argument for the renewed inclusion of EEG into the toolkit of developmental cognitive and social neuroscientists.

## 1 Introduction

Contemporary developmental cognitive neuroscience research has greatly advanced our understanding of the brain mechanisms underlying developmental trajectories from childhood to adulthood. There is now extensive and detailed knowledge of normative cognitive brain development, as well as extensive knowledge of atypical brain development and function. Neuroimaging studies predominantly use structural or functional magnetic resonance imaging (MRI) to study both brain structure and function. In the early stages of developmental cognitive neuroscience, seminal studies focused primarily on individual cognitive processes such as memory, attention, and language. More recently, there has been a movement toward incorporating social context into developmental cognitive neuroscience research (Beard et al., 2022, van Hoorn et al., 2019). This research extends beyond traditional cognitive domains and individual focus to study contextual and affective factors such as social relations and peer pressure. This field is sometimes referred to as developmental social neuroscience (Zelazo and Paus, 2010), in which social interactions are considered a variable of particular interest (see also Crone et al., this issue).

**Fig. 1:**
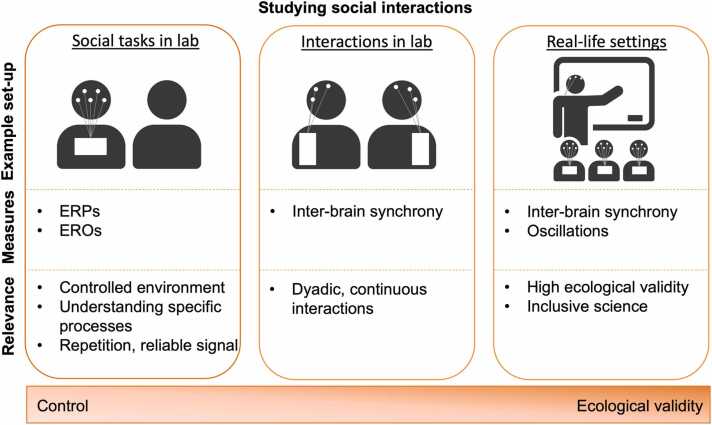
Overview of three novel research areas showing how EEG can be effectively used to study social interactions on a continuum from experimental control to ecological validity. Note: ERP = event-related potential; ERO = event-related oscillation.

As the neuroscience of social interactions gains increasing attention, especially in a developmental context, there is a need for innovative neuroscience methods that exhibit flexibility in measuring neural processes during social interactions. In addition, there is a growing need to improve the ecological validity of our laboratory paradigms (van Atteveldt et al., 2018). This includes conducting measurements in real-life settings, such as classrooms or homes, and during social interactions. In this perspective paper, we argue that EEG could be useful for measuring social aspects of neuroscience, particularly for developmental neuroscience. We will discuss examples from three separate research areas that study social interactions on a continuum from experimental control to ecological validity, from (implicit) social interaction tasks in the lab, via hyperscanning studies in the lab, to EEG studies in more naturalistic settings outside the lab (Fig. 1). We acknowledge that this continuum is an over-simplification, there are also examples of highly controlled paradigms that are also relatively high in ecological validity using representative design principles.

EEG research has a long history in the field of developmental cognitive neuroscience (see Norton et al., 2021 for more details). Broadly, EEG signals can be analyzed both in the time domain, by means of event-related potentials (ERPs), and in the frequency domain, by examining brain oscillations within specific frequency bands (e.g. alpha, beta, or theta) (Luck, 2014). Many EEG studies have examined ERP components in relation to developmental aspects. For example, in the context of this special issue, several researchers have investigated different aspects of cognitive control and attention in a developmental context (Overbye et al., 2018, Overbye et al., 2021). Other studies have examined developmental changes in the frequency domain. Typically, these studies focused on resting-state signals measured during a non-active baseline (e.g., with eyes closed). Resting state EEG signals can be reliable measures of cognitive function (Sargent et al., 2021) and markers of psychopathological states (Langer et al., 2022). Resting-state EEG oscillations have been successfully used to measure (cognitive) brain networks (Broyd et al., 2009, Mantini et al., 2007) and brain maturation (Smit et al., 2011), also in clinical populations (Janssen et al., 2017).

Another way to examine EEG signals is in the time-frequency domain. For example, event-related oscillations (EROs) allow for dynamic exploration of changes in specific frequency bands in response to certain events (Cohen, 2014). This transformation facilitates the identification of neurocognitive phenomena that may go unnoticed with traditional EEG analysis methods, such as ERPs (see Buzzell et al., 2022). Oscillations in specific frequency bands have been associated with specific cognitive mechanisms. For example, frontal theta oscillations have shown a robust association with cognitive control (Cavanagh and Frank, 2014, Hwang et al., 2016, van Noordt et al., 2022) and can be reliably assessed with a relatively small number of trials (Steele et al., 2016). There are several examples of successful ERO studies in relation to developmental stages (e.g., Malone et al., 2023; Morales et al., 2022; Tang et al., 2019) or as predictors of later psychopathology and substance use (Harper et al., 2021). These latter findings suggest that EROs may serve as sensitive biomarkers or endophenotypes of early alcohol exposure and other risk factors (Harper et al., 2018). However, it seems like developmental cognitive neuroscience has not yet fully embraced time-frequency analysis, so here we would like to argue that it is also useful for measuring social aspects of developmental neuroscience.

Other researchers, for example Buzzell et al. (2023) have already pointed out the various advantages of EEG, such as its excellent millisecond temporal precision. Moreover, EEG has a relatively low cost compared to alternative neuroscience techniques such as MRI and magnetoencephalography (MEG) (Buzzell et al., 2023, Luck, 2014). EEG offers less stringent exclusion criteria than fMRI, which can be particularly beneficial in developmental social neuroscience work. For example, people with braces or claustrophobia, who are typically excluded from MRI studies, can be included in EEG studies. Additionally, participants in EEG studies can sit instead of laying down in an MRI scanner, which also makes it more suitable for studying social interactions.

While EEG lacks the spatial resolution of fMRI and cannot capture structural brain development (Luck, 2014), it holds promise in certain areas such as social neuroscience. In this paper, we would like to highlight three separate research areas to provide examples of how EEG offers a unique added value to study social interactions. The first focuses on (implicit) social interaction tasks in the laboratory. Typically, in this type of study, two or more participants are *in the same room* and can observe each other or perform tasks together. A second relevant research area focuses on hyperscanning social interactions in the laboratory context, in which EEG is measured simultaneously from two (or more) participants. A third research area uses mobile EEG to measure brain activity outside the laboratory, in real-life settings.

## 2 Tasks with (implicit) social interaction in the EEG lab

EEG is an accessible tool for studying neural responses in social interactions. Participants can easily be in the same room and interact during the experiment while EEG is recorded (see Fig. 1). In this section, we summarize studies that measure EEG in one person while at least one other person is physically present. Although there are many ERP components relevant to social interaction, the majority of the studies we discuss here have looked at the process of performance monitoring or error monitoring. Performance monitoring is a crucial part of development as it can be an underlying process for observational learning (Yu and Zhou, 2006). The ERP component that is most often studied in relation to internal performance monitoring is the error-related negativity (ERN) (Falkenstein et al., 1991, Gehring et al., 1993). The ERN is a negative peak observed approximately 50–100 ms after the occurrence of an error. The ERN has shown to be modulated in social situations, for example, ERN amplitudes are increased when performance is evaluated by another participant (Hajcak et al., 2005, Voegler et al., 2018) or when participants make mistakes that harm others (de Bruijn et al., 2020). In contrast, the feedback-related negativity (FRN) is an ERP component studied in relation to external performance monitoring, as it is elicited in response to feedback related to inaccurate performance (Miltner et al., 1997).

In a study by Voegler and colleagues (2018) participants and patients with social anxiety disorder (SAD) performed a go/no-go task while being observed by another person. The results showed increased ERN amplitudes under social observation as compared to the control condition for both healthy controls and patients with SAD. Van Schie and colleagues (2004) showed that the ERN was not only increased by self-generated errors, but also by observing errors of someone else, suggesting that similar mechanisms are involved in monitoring one’s own errors and the errors of others.

De Bruijn and colleagues (2011) investigated the monitoring of one’s own and other’s errors in a cooperative context. Participants performed a social go/no-go task in pairs while EEG data was recorded from one of the participants. Their findings showed that when performing a task together, one incorporates the task of the other into one’s own error-monitoring process. This was reflected in an increased ERN following an error by one participant on compatible no-go stimuli only, thus when both participants had to inhibit their response, as compared to incompatible no-go stimuli (de Bruijn et al., 2011). Later, De Bruijn and Von Rhein (2012) showed a similar response-locked observation component in competitive contexts. These studies suggest that when observing other people perform a task, people covertly perform the task from their own perspective. Thus, comparing the goal action to the actual action performed by the participant, irrespective of the reward associated with the observed action.

Peterburs and colleagues (2019) used a similar version of a social go/no-go task in which participants and co-actors performed the task together. They differed between three types of trials: trials in which both had to respond, trials in which only the co-actor had to respond, and trials in which both the co-actor and the participants had to withhold their response. Instead of error monitoring, they focused on inhibition and attention/memory and their respective ERP components, the N2 and P3. Their findings showed a more negative N2 in trials in which both had to respond compared to the other trials, and a reduced P3b amplitude in trials in which both had to withhold compared to the other trials (Peterburs et al., 2019). Czeszumski and colleagues (2019) had a similar joint action set-up in which two participants actively perform a visual task either cooperatively or competitively, differentiating the two conditions by the nature of the rewards given (positive, negative, and neutral) to an error. The results showed the FRN to be more negative for losses than wins in both the cooperative and competitive context (Czeszumski et al., 2019).

Performance monitoring can also be affected in a potentially harmful social context, for example when our mistakes have negative consequences for other people. De Bruijn and colleagues (2020) investigated this in an experimental setting. Two participants were seated next to each other, one participant performed a version of the Eriksen flanker task (Eriksen and Eriksen, 1974) while EEG was recorded. In the harmful condition, the other participant would hear an aversive loud noise whenever the first participant made a mistake, while in the non-harmful context a soft, not unpleasant, beep was presented. The findings showed enhanced ERNs for harmful mistakes compared to non-harmful mistakes, suggesting a role of error significance in performance monitoring (de Bruijn et al., 2020). Jansen and De Bruijn (2020) continued this work by comparing mistakes harming the self, the other, and no one in individuals high and low in obsessive-compulsive symptoms (OCS). Individuals high in OCS showed enhanced ERNs for mistakes that harmed others instead of the self as compared to individuals low in OCS, who showed a decreased ERN for mistakes affecting no one versus oneself (Jansen and de Bruijn, 2020).

Another way of studying neural activation is through event-related oscillations (EROs). Buzzell et al. (2019) studied the effect of social observation on theta power in adolescents. Although the social condition did not involve two people physically present in the lab, they simulated social observation by using an online chatroom. Adolescents believed they were being observed by a peer in a chat room while doing a flanker task. In the social condition theta power was increased during error monitoring and proactive control. These findings support the moderating effect of social observation on adolescent cognitive control, reflected by increased to theta power (Buzzell et al., 2019).

Thus, EEG can easily be measured during social interactions in the laboratory and various performance monitoring processes measured by EEG may be affected by the social context. In particular, the ERN amplitude has shown to be enhanced when being observed or evaluated by others or when mistakes have negative consequences for another person, with an even higher enhancement in individuals high in OCS. Additionally, recent studies also pointed out other processes, such as reward processing and joint action to possibly be modulated in social contexts. Although studies within this field have mostly been conducted with adults, similar paradigms could be used to study social interactions during adolescence to investigate developmental aspects. This would be important, because social interactions are a crucial aspect of adolescence (Andrews et al., 2021, Blakemore, 2018, Blakemore and Mills, 2014).

## 3 Hyperscanning social interactions in the EEG lab

A novel method to study social interactions is EEG hyperscanning. Hyperscanning refers to the simultaneous recording of the brain activity of two or more people, while they are interacting with each other. It must be distinguished from the sequential recording of two subjects while exposed to the same stimulus (Hakim et al., 2023). Hyperscanning EEG studies often focus on inter-brain synchrony as a measure of inter-brain dynamics, which is the covariation of two brain signals in terms of phase, amplitude, frequency, or power. Inter-brain synchrony is not dependent on event-related designs that are necessary for ERP/ERO studies, allowing the use of more naturalistic paradigms, increasing ecological validity. This co-variation could be coincidental (due to a common external cause) or could derive from an information exchange between the two participants (Burgess, 2013). The literature presents a wide variety of inter-brain synchrony measures, each with its pros and cons, and a consensus on the best measure is still lacking (for an overview see Burgess, 2013; Czeszumski et al., 2020; Hakim et al., 2023).

During social interactions, more inter-brain synchrony is associated with improved behavioral coordination and communication (Dumas and Fairhurst, 2021, Pérez et al., 2019). Some studies have investigated the influence of the type of relationship between two people and their inter-brain synchrony during collaboration, and showed higher inter-brain synchrony in couples compared to friends and strangers (Djalovski et al., 2021, Kinreich et al., 2017).

EEG hyperscanning has been used to examine inter-brain dynamics in parent-infant dyads during unstructured social interaction. These naturalistic studies have related inter-brain dynamics to joint attention (Bánki et al., 2024), play (Wass et al., 2018), and emotional display (Perone et al., 2020, Santamaria et al., 2020). Leong and colleagues (2017) studied interbrain-synchrony also in infant-strangers dyads, comparing direct and averted gaze, in both live and screen mediated interaction (experimenter singing a nursery rhyme). They reported that direct eye-contact enhances adult-infant connectivity in both conditions (Leong et al., 2017).

EEG hyperscanning has also been applied in toddlers, children, and adolescents, both in the general population (Atzaba-Poria et al., 2017, Deng et al., 2023, Deng et al., 2024, Norton et al., 2022, Schwartz et al., 2022) and in clinical settings (Deng et al., 2022, Kang et al., 2023, Key et al., 2022, Samadani et al., 2021), using either structured (Deng et al., 2022, Deng et al., 2023, Deng et al., 2024) or naturalistic tasks (Atzaba-Poria et al., 2017, Kang et al., 2023, Key et al., 2022, Samadani et al., 2021, Schwartz et al., 2022). Using a picture processing task, Deng and colleagues (2024) observed that higher parental involvement (parental demonstration of interest in their child, caring, and warmth) is associated to higher central beta inter-brain synchrony in parent-adolescent dyads, when they experience positive emotions together. This suggests that parental involvement might enhance parent-adolescent emotional interaction. Using the same task, social anxiety was shown to increase or decrease inter-brain synchrony within a dyad, depending on the emotional state (positive or negative) of its members (Deng et al., 2022).

Key and colleagues (2022) studied inter-brain synchrony during a free-form friendly conversation between an autistic and a neurotypical adolescent. They reported that lower levels of synchrony during conversation are associated with increased social difficulties in the autistic adolescents (Key et al., 2022). These findings reinforce the idea that inter-brain coordination contributes to social behavior. Deng and colleagues (2023) investigated the effects of a mindfulness session on the inter-brain synchrony of adolescent dyads following a picture processing task. They observed that mindfulness significantly increases synchrony in central and frontal regions in the group that viewed different emotional stimuli together as compared to the non-mindfulness group (Deng et al., 2023).

The abovementioned studies have studied social interactions in children and adolescents, but have not focused on developmental aspects by comparing age groups or following children over time. Future longitudinal studies would be very important to understand the relationship between human brain development and social interaction. Only one study compared inter-brain synchrony during a collaborative computer game (structured task) between adolescents and young adults (Yang et al., 2023). Adolescents showed more inter-brain synchrony in delta and theta bands in the frontal, fronto-central and parietal regions, compared to young adults. The higher inter-brain synchrony observed in adolescent possibly reflects a higher perceived difficulty, which required them to employ more cognitive resources to achieve the same performance as the adult participants.

The introduction of hyperscanning represents an important step for bridging the gap between neuroscience and social and educational sciences. Particularly, hyperscanning represents a powerful tool to examine the neural underpinnings of social interaction and learning. Being an implicit and continuous measure, inter-brain synchrony could capture subtle and continuous changes in the interaction dynamics, which are not always visible at a behavioral level (Janssen et al., 2021, Tan et al., 2023). These studies on social interactions have shown increased inter-brain synchrony in closer relationships and lower levels of inter-brain synchrony in individuals experiencing difficulties with social interactions.

## 4 EEG in non-lab settings

Recent technical developments in mobile EEG make it possible to go a step further and measure brain activity outside the laboratory in real-life settings (Janssen et al., 2021, Stangl et al., 2023). Mobile EEG allows participants to freely move in the environment, wearing an EEG cap that is (wirelessly) connected to a small amplifier that can be attached to the participant. Using this novel technology, brain activity has been measured at home, at school, in a museum, or even outdoors. Mobile EEG studies typically focus on measures in the frequency domain (e.g. oscillations) that are not time-locked to stimuli, as it is difficult to present participants with enough ‘trials’ to compute ERPs in real-life settings. While being more naturalistic, which increases ecological validity, this type of studies comes at the cost of less experimental control.

Mobile EEG can be used in educational settings, for example studying reading, presentation patterns of learning materials, interactive behavior, edutainment, e-learning, motor skill acquisition, and promoting learning performance, focusing mostly on attention (Xu and Zhong, 2018). Although EEG recordings at school have been used to capture neural measures of attention in relation to instructional activities (Xu et al., 2022) and academic performance (Fuentes-Martinez et al., 2023), there is no consensus on the measures that are best suited to capture this. As this field is still in its infancy stage, various neural measures of attention have been used, such as alpha power and beta power spectral density (Fuentes-Martinez et al., 2023). Another outcome measure in EEG research in educational settings is inter-brain synchrony. More synchronized brain activity among students is related to engagement and social dynamics (Dikker et al., 2017), and learning (Davidesco et al., 2023). Additionally, increased inter-brain synchrony between teacher and students was related to better learning outcomes one week later (Davidesco et al., 2023). Another purpose of using mobile EEG in educational settings is to empower motivation and sense of control (Janssen and van Atteveldt, 2022). In the latter study, mobile EEG was used in a neurofeedback setup, in which adolescents experienced the control over their own brain activity in the context of a growth mindset intervention.

Finally, mobile EEG has also been used to measure brain activity when people are outside. For example, EEG has been used to record brain activity in different urban settings (Aspinall et al., 2015), when walking and sitting in a green space (Lin et al., 2020), and while navigating through a city (Wunderlich and Gramann, 2021). Other studies have adapted standard laboratory tasks (e.g. visual P300 paradigm, auditory oddball paradigm) to be administered while walking (Vařeka and Ladouce, 2021), doing an obstacle course (Reiser et al., 2019), and while cycling (Scanlon et al., 2019, Zink et al., 2016).

In addition to the benefits of ecological validity, it can also be argued that (mobile) EEG could make research more inclusive. EEG is easy to administer, so it can also be brought to communities that are usually underrepresented, for example people who typically do not participate in neuroimaging studies at a university but also to countries that lack proper infrastructure for conducting scientific research (Pietto et al., 2018, Williams et al., 2019). For example, in an interesting set of studies, Troller-Renfree et al., 2021, Troller-Renfree et al., 2022, Troller-Renfree et al., 2023 measured brain activity of infants born into households experiencing poverty. Administering EEG at home might also be beneficial for children with for example autism spectrum disorders, for whom it might be too stressful to visit a laboratory (Giannadou et al., 2022). Mobile EEG has also been combined with art to bring research to the general public and study real face-to-face interactions in museums and during concerts (Chabin et al., 2022, Dikker et al., 2021). These studies have shown that inter-brain synchrony during these interactions is related to empathy, social closeness, engagement, and social behavior (Dikker et al., 2021), to trait mindfulness (Chen et al., 2022a, Chen et al., 2022b), and to physical proximity and shared emotional state (Chabin et al., 2022).

Together, these studies show that it is possible to use mobile EEG to measure brain activity outside the laboratory in real-life settings. This is sometimes done in multiple participants at the same time (hyperscanning) but not necessarily. Increased inter-brain synchrony is related to closeness in social interactions and engagement in educational settings. This exciting field is relatively new, so there are still some challenges with for example data quality (lower signal-to-noise ration), intermittent loss of data during wireless transfer, and ethics (Janssen et al., 2021, Williamson, 2019). Additionally, more research is needed to clarify which methodologies and outcome measures can be used best, as these still vary considerably among studies (e.g. various frequency bands, inter-brain synchrony).

## 5 Discussion

Here we highlighted three separate research areas and provided examples of how EEG can be effectively used to study social interactions within a developmental cognitive neuroscience framework. First, lab-based EEG studies have shown that neural responses during performance monitoring (e.g. ERN) may be specifically affected by the physical presence of and interaction with others (De Bruijn and von Rhein, 2012, Hajcak et al., 2005, van Schie et al., 2004, Voegler et al., 2018). Second, EEG can be measured simultaneously in two or more people in hyperscanning studies to dive deeper into the interactive processes. These studies have shown that inter-brain synchrony is higher in closer relationships (Djalovski et al., 2021, Kinreich et al., 2017) and might be influenced by difficulties with social processing (e.g. in people with autism spectrum disorders (Key et al., 2022) or social anxiety symptoms (Deng et al., 2022)). Third, mobile EEG provides the opportunity to take this research outside the laboratory to make research more inclusive and to study neural activity in real-life settings. These studies have shown that higher inter-brain synchrony is related to more engagement and higher performance in students (Davidesco et al., 2023, Dikker et al., 2017) . We believe that future research would benefit from applying a developmental perspective and more integration of these three separate research areas with different levels of ecological validity and experimental control.

These three research areas together form a continuum from studying social interactions with greater experimental control (in lab-based studies) to more ecological validity (in real-life settings; see Fig. 1). These different types of studies strengthen one another by generating new questions and hypotheses and should thus be interconnected in a cyclic manner (from lab to real-life and back to the lab; Matusz et al., 2019). The introduction of EEG hyperscanning and mobile EEG represents an important step for bridging the gap between neuroscience and educational and social sciences. These are powerful tools to examine the neural underpinnings of social interactions, since it allows to record brain activity in real-life settings, as the action unfolds (Janssen et al., 2021, Tan et al., 2023). As such, inter-brain synchrony could capture subtle and continuous changes in social interactions, which are not always visible at a behavioral level. On the other side, there are also challenges in these EEG studies with more ecological validity, such as greater likelihood of movement artifacts, lack of alignment in EEG frequency bands over age, and few data processing pipelines for developmental hyperscanning. Connecting EEG studies with high ecological validity with EEG lab studies with high experimental control might help overcome some of these challenges.

Given the scarcity of naturalistic studies targeting inter-brain synchrony in social interactions from a developmental perspective, we think that more research on this topic would be crucial. In fact, children’s brain and behavior are not only shaped by the relationship with parents, but also with peers. This holds even truer during adolescence, when the time spent with peers increases, together with the desire for social acceptance and the importance given to peers’ opinions and expectations (Brown and Larson, 2009). Adolescence is also characterized by a maturation of socio-cognitive capacities, such as theory of mind and perspective-taking, which are fundamental for social interactions (Valle et al., 2015). Therefore, it would be important to study the neural mechanisms underlying these social interactions.

Inter-brain synchrony is still relatively new and some mechanisms of inter-brain synchrony are still unclear. For example, it is still debated whether inter-brain synchrony during social interaction is simply an epiphenomenon, derived from synchronous motor activity or entrainment to the same upcoming external stimuli (Holroyd, 2022), or whether it also reflects a deeper connection, based on internal mental and emotional states (Dikker et al., 2021). Moreover, the current methods cannot establish whether higher inter-brain synchrony is a consequence or a cause of more synchronized behavior (Holroyd, 2022, Novembre et al., 2023, Novembre and Iannetti, 2021). Multibrain stimulation with transcranial magnetic stimulation or transcranial alternating current stimulation might be a way to prove the causal relationship between inter-brain synchrony and behavior (Novembre et al., 2023, Novembre and Iannetti, 2021). Some promising results have been recently obtained through multibrain stimulation, indicating that the induction of inter-brain synchrony increases movement synchrony during finger tapping (Novembre et al., 2017), improves communication (Chen et al., 2022a, Chen et al., 2022b) and social learning (Pan et al., 2021). So, future research should clarify the mechanisms underlying inter-brain synchrony and its relationship with behavior.

In this perspective we only focused on EEG for clarity, but we acknowledge that other neuroimaging techniques ((f)MRI, fNIRS, TMS, MEG) are also important in developmental cognitive neuroscience. Each technique has its own advantages and disadvantages, and the best method differs per research question. Also, it should be noted that there are also some disadvantages of EEG. Putting an EEG cap on takes time and might be a bit uncomfortable, EEG is limited to recording of brain activity on the surface (Luck, 2014), and the signal is sensitive to motion artifacts (e.g. more than e.g. fNIRS) (Czeszumski et al., 2020). Moreover, several repetitions of each event of interest are needed (Luck, 2014), which increases task duration and decreases ecological validity. Recent technical advances (such as described in an earlier special issue of Developmental Cognitive Neuroscience introduced by Buzzell et al. 2023) can provide solutions for some, but not all, of these challenges. Nevertheless, EEG’s excellent temporal precision, ease of use, relatively low cost, and its usefulness to measure social interactions make us argue that EEG is an important tool for developmental cognitive neuroscience.

Given the growing emphasis on larger longitudinal cohort studies in developing populations and these exciting new developments, we also argue for the integration of EEG as a valuable tool in large-scale developmental cognitive neuroscience. This integration can contribute to mapping functional brain development across developmental stages, providing more insight on the relationship between brain development and social interactions, and to generalizing results beyond Western, educated, industrialized, rich, and democratic (WEIRD) populations. Despite the aforementioned advantages, it remains somewhat puzzling why EEG is not commonly included in large-scale cohort studies. There is currently a growing imperative to utilize larger sample sizes in neuroscience studies to increase the reliability and precision of research findings, thus increasing confidence in the results (Button et al., 2013, Marek et al., 2022). In recent years, the inclusion of (f)MRI in these large-scale, often multicenter, developmental cognitive neuroscience studies has led to significant advances in our understanding of the field, as evidenced by initiatives such as the Adolescent Brain Cognitive Development (ABCD) study (Feldstein Ewing et al., 2018) the Imagen study (Schumann et al., 2010), and the Generation R study (see White et al., 2018 for a review). To our knowledge, there are only two large-scale studies that include EEG: the Minnesota Twin Family Study (Iacono et al., 1999) and the Healthy Brain and Child Development (HBCD) study (Norton et al., 2021). A landmark investigation within the Minnesota study revealed that EEG P3 amplitude serves as a prospective predictor of problematic alcohol use later in life, potentially serving as a biomarker for alcohol use disorders (AUDs) (Iacono et al., 1999).

In the GUTS consortium (Crone et al., this issue), we are including EEG measures to assess social interactions in a large cohort of children and adolescents. We will follow these children over 10 years to examine the role of self-regulation in how these children grow up to be contributing members of society. As self-regulation is a very broad concept, we will focus on three processes in our EEG study: reward processing (Williams et al., 2021), error processing (Barker et al., 2018, Buzzell et al., 2017, Eriksen and Eriksen, 1974), and inhibitory control (Pfefferbaum et al., 1985). Additionally, we will focus on social contexts, by comparing rewards for self versus somebody else and by hyperscanning children to study observational effects on error processing, and on associations with both internalizing and externalizing symptoms.

In sum, given the specific benefits of measuring EEG in a social context, we call for a renewed focus on this measure in the field of developmental cognitive neuroscience. We summarize some exciting studies on (implicit) social interaction tasks in the lab, hyperscanning social interactions in the lab, and mobile EEG in real-life settings. These research areas form a continuum from experimental control to ecological validity and strengthen each other in elucidating the important role of social interactions across development.

## CRediT authorship contribution statement

**Ingmar Franken:** Writing – review & editing, Writing – original draft, Conceptualization. **Tieme Janssen:** Writing – review & editing, Writing – original draft. **Laura Fornari:** Writing – review & editing, Writing – original draft. **Nienke van Atteveldt:** Writing – review & editing, Writing – original draft. **Ellen R. A. de Bruijn:** Writing – review & editing. **Anita Harrewijn:** Writing – review & editing, Writing – original draft, Conceptualization. **Yvette Grootjans:** Writing – review & editing, Writing – original draft, Conceptualization.

## Declaration of Competing Interest

The authors declare that they have no known competing financial interests or personal relationships that could have appeared to influence the work reported in this paper.
